# Novel Point-of-Care Diagnostic Method for Neonatal Encephalopathy Using Purine Nucleosides

**DOI:** 10.3389/fnmol.2021.732199

**Published:** 2021-09-09

**Authors:** Edward Beamer, Mary Isabel O’Dea, Aisling A. Garvey, Jonathon Smith, Aida Menéndez-Méndez, Lynne Kelly, Andreea Pavel, Sean Quinlan, Mariana Alves, Eva M. Jimenez-Mateos, Faming Tian, Eugene Dempsey, Nicholas Dale, Deirdre M. Murray, Geraldine B. Boylan, Eleanor J. Molloy, Tobias Engel

**Affiliations:** ^1^Department of Physiology and Medical Physics, Royal College of Surgeons in Ireland, University of Medicine and Health Sciences, Dublin, Ireland; ^2^Centre for Bioscience, Manchester Metropolitan University, Manchester, United Kingdom; ^3^Coombe Women and Infants University Hospital, Dublin, Ireland; ^4^National Children’s Research Centre, Crumlin, Dublin, Ireland; ^5^Discipline of Paediatrics, Children’s Health Ireland at Crumlin and Tallaght, Dublin, Ireland; ^6^Trinity Research in Childhood Centre (TRiCC), Trinity College Dublin, The University of Dublin, Dublin, Ireland; ^7^INFANT Research Centre, University College Cork, Dublin, Ireland; ^8^Department of Paediatrics and Child Health, University College Cork, Dublin, Ireland; ^9^FutureNeuro, Science Foundation Ireland Research Centre for Chronic and Rare Neurological Diseases, Royal College of Surgeons in Ireland, University of Medicine and Health Sciences, Dublin, Ireland; ^10^Discipline of Physiology, School of Medicine, Trinity College Dublin, The University of Dublin, Dublin, Ireland; ^11^School of Life Sciences, University of Warwick, Coventry, United Kingdom

**Keywords:** neonatal encephalopathy, biomarker, purines, mouse models, clinical testing, seizures

## Abstract

**Background:** Evidence suggests that earlier diagnosis and initiation of treatment immediately after birth is critical for improved neurodevelopmental outcomes following neonatal encephalopathy (NE). Current diagnostic tests are, however, mainly restricted to clinical diagnosis with no molecular tests available. Purines including adenosine are released during brain injury such as hypoxia and are also present in biofluids. Whether blood purine changes can be used to diagnose NE has not been investigated to date.

**Methods:** Blood purines were measured in a mouse model of neonatal hypoxia and infants with NE using a novel point-of-care diagnostic technology (SMARTChip) based on the summated electrochemical detection of adenosine and adenosine metabolites in the blood.

**Results:** Blood purine concentrations were ∼2–3-fold elevated following hypoxia in mice [2.77 ± 0.48 μM (Control) vs. 7.57 ± 1.41 μM (post-hypoxia), *p* = 0.029]. Data in infants with NE had a 2–3-fold elevation when compared to healthy controls [1.63 ± 0.47 μM (Control, *N* = 5) vs. 4.87 ± 0.92 μM (NE, *N* = 21), *p* = 0.0155]. ROC curve analysis demonstrates a high sensitivity (81%) and specificity (80%) for our approach to identify infants with NE. Moreover, blood purine concentrations were higher in infants with NE and seizures [8.13 ± 3.23 μM (with seizures, *N* = 5) vs. 3.86 ± 0.56 μM (without seizures, *N* = 16), *p* = 0.044].

**Conclusion:** Our data provides the proof-of-concept that measurement of blood purine concentrations via SMARTChip technology may offer a low-volume bedside test to support a rapid diagnosis of NE.

## Introduction

Neonatal encephalopathy (NE), characterized by disturbed neurological function in infants born ≥ 35 weeks of gestation, is considered one of the most serious birth complications of full-term infants accounting for 23% of infant mortality worldwide (Millar et al., 2017). Infants with NE who survive infancy have an increased risk of developing life-long neurological disorders (e.g., learning disabilities, cerebral palsy, autism, epilepsy), which adds significantly to the burden of NE (Shetty, 2015). The combination of hypoxia and ischemia is commonly associated with NE regardless of etiology (Aslam et al., 2019; Chalak et al., 2019). Therapeutic hypothermia (TH) initiated within 6 h of birth is the only standard-of-care treatment for moderate to severe NE (Wassink et al., 2019). Despite this, long-term disabilities persist in 50% of infants (Jacobs et al., 2013).

Evidence suggests that the key to better neurodevelopmental outcomes following NE is earlier diagnosis and initiation of TH immediately after birth (Thoresen et al., 2013). Nevertheless, reliable and early diagnosis of NE remains a clinical challenge. Current criteria for NE diagnosis such as Apgar scores, need for delivery room intubation/resuscitation, blood pH and neurological signs may sometimes be inaccurate, inconsistent or misleading (Robertson and Perlman, 2006). Early electroencephalogram (EEG) monitoring is useful to aid in diagnosis but requires expertise and specialist equipment for interpretation, which is not always available in the Neonatal Intensive Care Unit (NICU), along with a high degree of associated costs (Murray et al., 2016). There is, therefore, a pressing need for the development of new, reliable diagnostic and prognostic tests that will help accurately identify cases of NE as soon as possible after birth to guide the initiation of treatment and predict clinical outcomes. Several circulating biomarkers are currently under investigation (Graham et al., 2018). Among these, alterations in microRNA levels in umbilical cord blood (O’Sullivan et al., 2019) and elevations of pro-inflammatory cytokines in the blood (Chaparro-Huerta et al., 2017; Omer et al., 2020; Sweetman et al., 2020) have shown promising results. Detection techniques for these molecules do not support a fast diagnosis, requiring sample preparation and sophisticated equipment [e.g., enzyme-linked immunosorbent assays (cytokines) and qPCR (microRNA)], potentially delaying the diagnosis past the ideal therapeutic window of < 6 h post-delivery. Thus, there remains a need for a molecular biomarker of NE to enable a fast, affordable detection in an easy-to-use point-of-care device.

Purinergic signaling, characterized as a complex regulatory system governed by purine nucleotides such as ATP and nucleosides, is involved in many critical processes of brain development, such as cell proliferation and differentiation, neuron-glia communication, neurotransmitter modulation and inflammatory processes (Rodrigues et al., 2019). In animal models of NE, inflammatory signaling is enhanced in conjunction with upregulation of purine receptors, which can be targeted therapeutically to reduce pathology (Rodriguez-Alvarez et al., 2017; Menendez Mendez et al., 2020). Normally, purine signaling molecules (e.g., ATP, adenosine) have relatively low extracellular concentrations, and are also actively released following cellular stress such as inflammation and hypoxia (Takahashi et al., 2010; Thauerer et al., 2012). Blood purine levels (adenosine and adenosine metabolites) have been shown to be increased in the blood following stroke, ischemic brain injuries (Saugstad, 1975; Weigand et al., 1999; Laghi Pasini et al., 2000; Tian F. et al., 2017; Dale et al., 2019) and, as shown more recently, during epilepsy (Beamer et al., 2021). Increases in purine nucleoside concentrations in biofluids post-hypoxia have been reported previously in experimental models of neonatal hypoxia suggesting diagnostic potential of purines for NE (Kuligowski et al., 2017) and also in infants following hypoxia [e.g., increased purines levels in cerebro-spinal fluid (CSF) (Harkness and Lund, 1983) and plasma (Saugstad, 1975; Buonocore et al., 2000)]. However, purines were not favorable as a diagnostic tool due to their short half-life and by being difficult to measure. Recently a novel enzyme-based amperometric biosensor (SMARTChip) was demonstrated to detect a summed concentration of the purine nucleoside adenosine and its metabolites, xanthine, hypoxanthine and inosine (Dale and Frenguelli, 2009). Increases in purine nucleoside concentration measured using this tool from whole blood can offer a fast and reliable diagnostic tool for ischemic stroke in adults (Dale et al., 2019), and can distinguish epileptic patients from healthy individuals (Beamer et al., 2021). Here, we investigated whether this approach was also useful in the detection of NE. Using a mouse model of neonatal hypoxia (Rodriguez-Alvarez et al., 2015) and measurements taken from infants with NE, we investigated whether the SMARTChip technology could reliably detect a spike in blood concentration of purine nucleosides associated with NE.

## Materials and Methods

### SMARTChip and Blood Purine Measurement

**FIGURE 1:**
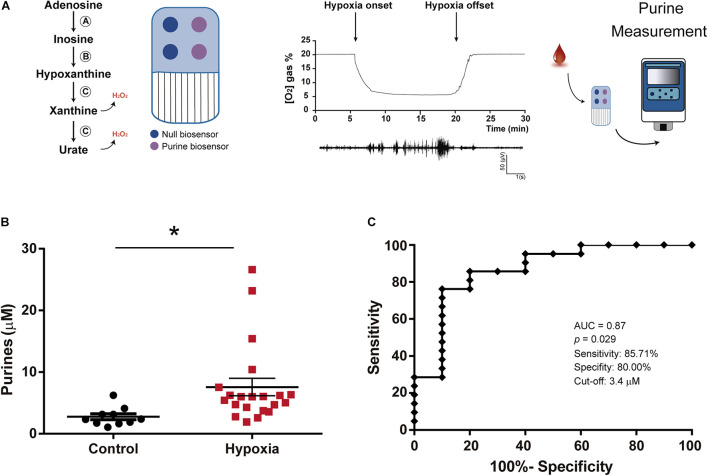
Increased blood purine concentrations following hypoxia in mice. **(A)** Schematic outlining functioning of SAMRTChip and neonatal hypoxia model in mice. Blood purines are detected via an enzymatic cascade with enzymes entrapped within a layer on a Ruthenium Purple-coated gold electrode. This includes (A) Adenosine deaminase which converts adenosine into inosine, (B) Nucleoside phosphorylase which converts inosine into hypoxanthine and (C) Xanthine oxidase which converts hypoxanthine into xanthine and H_2_O_2_ and subsequently xanthine into urate and H_2_O_2_. Each SMARTchip consists of two purine biosensors and two null biosensors. Final read is the difference between purine sensors subtracting the background measured via null sensors. P7 mice are subjected to 15 min of 5% O_2_ experiencing hypoxia-induced seizures as detected via EEG recording. Blood purine concentrations were measured immediately following hypoxia using the SMARTChip. **(B)** Graph showing increased purine concentrations in blood post-hypoxia in mouse pups. Whole trunk blood samples were collected between 0 and 10 min post-hypoxia (*N* = 10 control and 21 hypoxia), Unpaired Student’s *t*-test (*t* = 2,296; *df* = 29; *p* = 0.029). **(C)** ROC analysis shows blood purine concentration had a 85.71% sensitivity, 80.00% specificity and AUC of 0.87 for identifying mice with hypoxia at a cut-off of 3.4 μM (*N* = 10 control and 21 hypoxia). *p < 0.05.

The function of the SMARTChip (Sarissa Biomedical, Coventry, United Kingdom) has previously been described in detail (Tian D.S. et al., 2017; Dale et al., 2019; Beamer et al., 2021). Briefly, a gel matrix is anchored to a gold-plated electrode and contains a layer of three enzymes allowing for the detection of adenosine, inosine, hypoxanthine and xanthine (Figure 1A). Enzymes include: (1) Adenosine deaminase converting adenosine into inosine, (2) Nucleoside phosphorylase converting inosine into hypoxanthine and, (3) Xanthine oxidase converting hypoxanthine into xanthine and H_2_O_2_ and urate and H_2_O_2_. Amperometric measurement allows then for the detection and quantification of electroreduction via peroxide produced by Xanthine oxidase. This iterative sequence of enzymatic reactions will lead to the formation of electroactive hydrogen peroxide which is proportional to the summative concentration of all four purines (adenosine and the intermediate substrates, xanthine, hypoxanthine, and inosine) in the blood. Each SMARTChip features two purine and two null sensors. Null sensors lack sensitivity to purines, thereby acting as a control for any non-specific interferences. All measurements are performed as the difference between the two biosensors and the two null electrodes giving a total of four reads for each sample: [(Purine sensor 1)—(Null sensor 1), (Purine sensor 1)—(Null sensor 2), (Purine sensor 2)—(Null sensor 1), (Purine sensor 2)—(Null sensor 2)]. The final result for each sample is the average of all four reads. Before blood measurements, each SMARTChip is calibrated in a solution containing 10 μM adenosine buffer solution (adenosine in H_2_O), ensuring thereby minimal variability between sensors. Accuracy and sensitivity of SMARTChips to adenosine and adenosine down-stream purines (xanthine, hypoxanthine and inosine) have been verified previously (Tian F. et al., 2017; Beamer et al., 2021). Moreover, previous studies using SMARTChip detection technology found very little interference with purine measurement for ascorbate, acetaminophen and urate (Beamer et al., 2021) which collectively provide more than 97% of the interfering signal for electrochemical measurements (Albery et al., 1993). Measurements were taken from whole blood taken from mice or infants, which was immediately placed onto the gel-matrix-coated electrode and, results were produced within minutes.

## Neonatal Mouse Model of Hypoxic NE

All procedures were performed in accordance with the guidelines of the European Communities Council Directives (86/609/EU and 2010/63/EU) and were reviewed and approved by the Research Ethics Committee of the Royal College of Surgeons in Ireland (RCSI) (REC1302b) under license from the Department of Health, Dublin, Ireland (B100/4524). Litters of male and female C57BL/6 OlaHsd mice (Harlan, United Kingdom) were kept with their dams in a barrier-controlled facility on a 12 h light-dark cycle with access to food and water *ad libitum*. Hypoxia was induced as described by exposing P7 mice (weight 4–6 g), a developmental stage that roughly equates to the age of a term infant (Semple et al., 2013), to a 95% N_2/_5% O_2_ premixed gas for 15 min. Normoxic controls were placed in the chamber at 21% O_2_ for the same period of time. Electrodes were implanted in both experimental groups (normoxic controls and pups subjected to hypoxia) and EEG recorded as previously described to confirm the presence of seizures (Rodriguez-Alvarez et al., 2015). Hypoxic conditions, 5% oxygen concentrations, were confirmed using the Pico2-OEM optical oxygen meter (PyroScience, Germany). A droplet of trunk blood was taken immediately following 15 min of hypoxia to measure purine nucleoside concentrations using the SMARTChip assay.

## Newborn Patient Data and Clinical Measurements

This study was approved by the Ethics Committee of the Coombe Women and Infants University Hospital (CWIUH), Dublin, Ireland, and Clinical Research Ethics Committee of the Cork Teaching Hospitals (CUMH) [ECM 5(5) 04/07/17], Cork, Ireland, both tertiary NICUs, and national referral centers for TH. Families received verbal and documented information on the study and written consent was obtained prior to recruitment. We have previously recruited several distinct cohorts of infants with NE and controls with criteria as described (O’Hare et al., 2017; Sweetman et al., 2019). The severity of NE was classified by Sarnat Staging (Sarnat and Sarnat, 1976). Infants with congenital abnormalities or evidence of maternal substance abuse were excluded. Whole blood sampling was performed following informed parental consent. Sampling was performed using aseptic technique via central and peripheral arterial lines and via venous sampling at times of routine patient phlebotomy and processed immediately. The volume taken was approved by the Ethics committee, as a volume that would not affect the infant’s hemodynamic status (approximately 20–50 μl). Neonatal seizures were diagnosed (*N* = 5) using a combination of clinical signs and amplitude integrated electroencephalography (aEEG) or continuous EEG (cEEG). One infant died. Brain Magnetic resonance imaging (MRI) was performed after completion of TH on days 5–10 of life as part of routine clinical care. Our study included 21 patients with NE and graded as follows: Sarnat score I (*N* = 3); Sarnat score II (*N* = 15); Sarnat score III (*N* = 3). Blood was taken as early as possible in order to test the potential of stratification of infants according to severity via an arterial line, from time of delivery until 4 days post-delivery from indwelling arterial or venous umbilical catheters in neonates undergoing TH. Infants with mild NE did not receive TH. Healthy term aged-matched controls included asymptomatic infants (*N* = 5, 40% female) with an uneventful delivery, normal Apgar scores, normal neonatal examinations, and without admission to the NICU who underwent routine phlebotomy following informed consent. Infants undergoing sepsis evaluations or receiving phototherapy for jaundice were excluded. Blood was taken 2–3 days post-delivery via peripheral venous sampling (details in Supplementary Table 1).

## Statistical Analysis

Statistical analysis of data was performed using Prism 8 (GraphPad) and STATVIEW software (SAS Institute). Data are mean ± standard error of the mean (SEM). Unpaired Student’s *t*-test was used for two-group comparison. Mann Whitney test was performed to compare differences between two independent groups where the dependent variable was either ordinal or continuous, but not normally distributed. Receiver Operator Characteristic (ROC) analysis was performed to investigate the diagnostic ability of purine measurements for hypoxia in mouse pups and infants with NE. Correlations between variables were assessed using Pearson’s correlation coefficient. Significance was accepted at *p* < 0.05.

## Results

### Increased Blood Purine Concentrations Following Hypoxia in Mice

Using a mouse model of neonatal hypoxia (Rodriguez-Alvarez et al., 2015), we tested whether neonatal hypoxia leads to an increase in blood purine concentrations. Blood purines were measured via SMARTChip in whole blood in P7 mouse pups subjected to hypoxia or normoxia (Control) conditions for 15 min at the time of re-oxygenation (Figure 1A). This analysis provides a quantitative and summative analysis of adenosine and the adenosine downstream purines inosine, hypoxanthine and xanthine (Dale et al., 2019; Beamer et al., 2021). Hypoxia-induced seizures were confirmed via cranial EEG recordings (Figure 1A). Mouse pups subjected to hypoxia showed a ∼2-3 fold increase in purine nucleosides when compared to normoxic control mice [2.77 ± 0.48 μM (Control, *N* = 10) vs. 7.57 ± 1.41 μM (post-hypoxia, *N* = 21), *p* = 0.029] (Figure 1B). ROC curve analysis demonstrated that changes in blood concentrations of purines have a high level of sensitivity (85.71%) and specificity (80.00%) to discriminate between control mice and mice subjected to hypoxia at a cut-off of 3.4 μM with an area under the curve (AUC) of 0.87 (Figure 1C). Thus, our data demonstrates that hypoxia in mouse pups leads to changes in blood purine levels and that we can detect these increases by using our SMARTchip technology.

### Increased Blood Purine Concentrations Following NE in Infants

**FIGURE 2:**
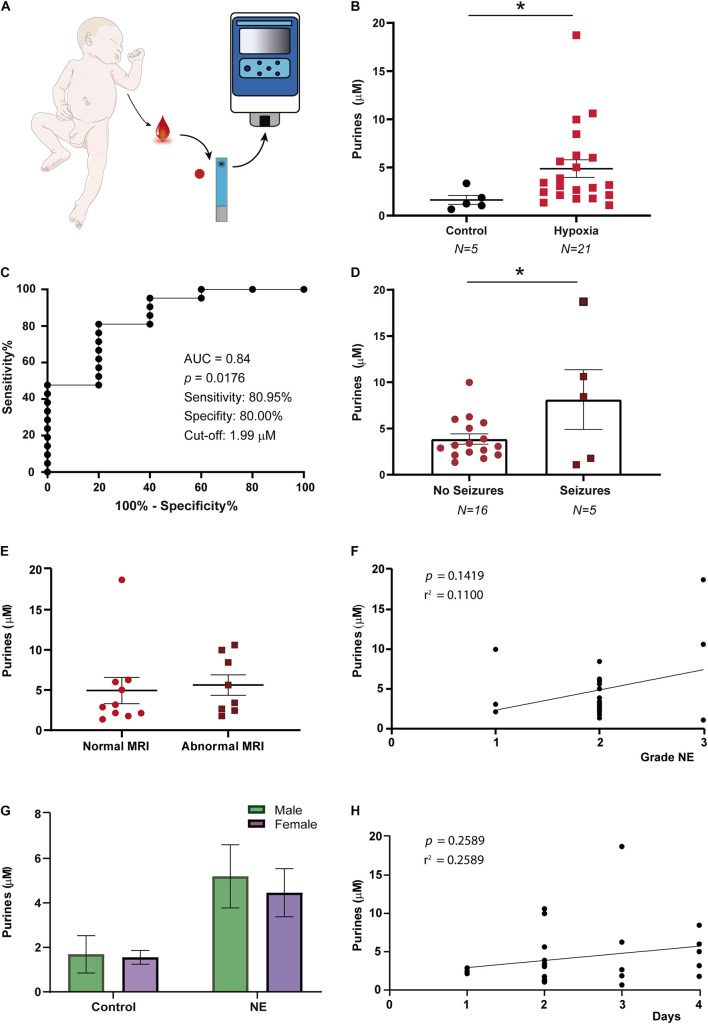
Purine concentration changes in infants with neonatal encephalopathy. **(A)** Blood purine concentration was analyzed in whole blood via SMARTchip 1–4 days post-delivery. **(B)** Increased blood purine concentrations in infants with NE (*N* = 15) when compared to healthy controls (*N* = 5), Mann-Whitney test (*p* = 0.0155). **(C)** ROC analysis shows blood purine concentration had a 80.95% sensitivity, 80.00% specificity and AUC of 0.84 for identifying infants with NE at a cut-off of 1.99 μM. **(D)** Graph showing blood purine concentrations measured in infants with NE are higher in the presence of seizures (*N* = 16 (NE without seizures) and 5 (NE with seizures), Unpaired Student’s *t*-test (*t* = 2.160; df = 19; *p* = 0.044). **(E)** Graph showing no correlation between purine concentrations when comparing infants with normal MRI (*N* = 10) with infants with an abnormal MRI (*N* = 8) (*p* = 0.7572). **(F)** No correlation between purine concentrations and severity of NE (*N* = 21, *p* = 0.1419). **(G)** No differences in purine concentrations between male and female infants under control conditions (male, *N* = 3; female, *N* = 2) (*p* = 0.9991) and following NE (male, *N* = 12; female, *N* = 9; *p* = 0.8982). **(H)** No correlation between the time point of blood sampling and purine blood concentrations in infants with NE (*N* = 26, *p* = 0.26). *p < 0.05.

Clinical testing in infants (*N* = 26) (Figure 2A and Table 1) revealed a ∼2–3-fold increase in blood concentration of purine nucleosides following NE [1.63 ± 0.47 μM (Control, *N* = 5) vs. 4.87 ± 0.92 μM (NE, *N* = 21), *p* = 0.0155; Figure 2B], similar to our results obtained in mouse pups subjected to hypoxia. ROC curve analysis showed that the SMARTChip assay has a high level of sensitivity (80.95%) and specificity (80.00%) for detecting NE in neonates at a cut-off of μM 1.99 with an AUC of 0.84 (Figure 2C). Previous data, using the same detection technology, has suggested seizures lead to increases in blood purine concentrations in mice (Beamer et al., 2021). Therefore, to test whether neonatal seizures increase purine levels even further, infants with NE were grouped according to the presence or absence of seizures. This analysis revealed that the presence of neonatal seizures was associated with higher blood concentrations of purines [3.86 ± 0.56 μM (NE without seizures, *N* = 16) vs. 8.13 ± 3.23 μM (NE with seizures, *N* = 5), *p* = 0.044; Figure 2D]. No correlation was, however, found between blood purine concentrations and abnormal MRI (*p* = 0.76) (Figure 2E) and Apgar score (*p* = 0.1419) (Figure 2F). Also, no differences in blood purine levels were observed between male and female infants under control conditions and post-NE [Control: 1.69 ± 0.84 μM (male, *N* = 3) vs. 1.55 ± 0.31 μM (female, *N* = 2) and NE: 5.2 ± 1.42 μM (male, *N* = 12) vs. 4.6 ± 1.1 μM (female, *N* = 9)] (Figure 2G) and the time of blood sampling (*p* = 0.26) (Figure 2H). Taken together, our results demonstrate the potential of our SMARTChip technology in measuring blood purine levels as an informative clinical tool to aid in the detection of NE.

**TABLE 1:** Clinical details of infants with corresponding blood purine concentrations.

ID	Hospital	Grade of encephalopathy	Sex	Time of measurement (day of life)	Seizures	Purines (μM)
1	CWIUH	1	F	2	No	3.07
2	CWIUH	2	M	4	Yes	1.78
3	CWIUH	2	M	1	No	2.14
4	CWIUH	2	M	1	No	2.88
5	CWIUH	2	M	4	No	3.18
6	CWIUH	2	M	2	No	3.88
7	CWIUH	2	M	4	No	5.02
8	CWIUH	2	M	2	No	5.62
9	CWIUH	2	M	4	No	6.00
10	CWIUH	2	F	3	No	6.25
11	CWIUH	2	F	4	Yes	8.45
12	CWIUH	3	F	2	Yes	10.61
13	CWIUH	3	M	3	Yes	18.72
14	CUMH	1	F	1	No	2.12
15	CUMH	1	M	2	No	9.98
16	CUMH	2	M	2	No	1.35
17	CUMH	2	M	2	No	1.76
18	CUMH	2	F	1	No	2.44
19	CUMH	2	F	3	No	2.66
20	CUMH	2	F	2	No	3.41
21	CUMH	3	F	2	Yes	1.09
22	CWIUH	Control	M	2	−	1.05
23	CWIUH	Control	M	3	−	0.66
24	CWIUH	Control	M	2	−	3.35
25	CWIUH	Control	F	3	−	1.24
26	CWIUH	Control	F	3	−	1.86

*CWIUH, Coombe Women and Infants University Hospital (Dublin, Ireland); CUMH, Cork Teaching Hospitals (Cork, Ireland).*

## Discussion

This is the first demonstration of a simple method for the detection of increased blood purine levels in infants with NE at the bedside, which could provide a novel tool to support an early and fast diagnosis of infants with NE. Currently, there is no single gold standard biochemical biomarker to diagnose NE nor prognosticate severity with accuracy in clinical use. Brain MRI can help determine occurrence of injury, but is only possible between 7 and 10 days after birth, i.e., outside the therapeutic window. Circulating inflammation makers such as cytokines (Chaparro-Huerta et al., 2017; Omer et al., 2020; Sweetman et al., 2020) or microRNAs (O’Sullivan et al., 2019), have shown great promise as novel diagnostic tools to support the identification of infants with NE. Analyzing these markers requires, however, sample preparation and, in some cases, equipment which is not suited for use in the NICU. While it is well established that hypoxia leads to changes of purines in biofluids (Saugstad, 1975; Harkness and Lund, 1983; Buonocore et al., 2000), their instability in biofluids and the need for sophisticated equipment [e.g., high-performance liquid chromatography (HPLC)] has hamperd their progression as biomarkers into the clinic. Here, we report increased blood purine levels in mice subjected to hypoxia and in infants with NE using a new technology, SMARTChip. This technique employs an electrochemical method for a rapid detection of purines in whole, unprocessed blood via a user-friendly diagnostic device (Dale et al., 2019), thereby overcoming several limitations of current biomarkers under investigation for NE.

Our results also show that blood purine levels increase further in neonates with NE when seizures are present. The prognosis for infants with NE and seizures is particularly poor, with seizures leading to an increased rate of mortality and worsening of clinical outcomes (Pisani and Spagnoli, 2016). EEG is an excellent tool for assessing the severity of NE and for detecting seizures but interpretation in neonates is an ongoing challenge requiring specialized personnel and equipment (Boylan et al., 2015). Our results suggest that analyzing blood purine levels may not only support the detection of NE, but may also help in the identification of infants at risk of neonatal seizures. This would be in good agreement with a previous study showing increased blood purine concentrations following provoked seizures in mice (Beamer et al., 2021). It is tempting to speculate, therefore, that the elevated blood purine levels in infants with NE and seizures are a combination of both hypoxia- and seizure-driven increases in purine release. It is, however, also possible that the additional increases are simply the result of a more severe NE which is usually accompanied by seizures (Pisani and Spagnoli, 2016).

Importantly, purine concentrations measured in this study (low μM concentration range) are similar to previous studies using the electrochemical detection approach (i.e., SMARTChip) (Dale et al., 2019; Beamer et al., 2021) or studies using other detection techniques (e.g., HPLC) (Harkness and Lund, 1983; Zielinski et al., 2019). Moreover, blood purine concentrations at baseline and blood purine increases under pathological conditions (i.e., hypoxia in mice and NE in neonates) are similar between species, demonstrating its translatability from animal models to patients.

ATP is widely known to be released from cells throughout the body under hypoxic stress, and is rapidly metabolized into purine nucleosides. In fact, adenosine release allows tissue adaption under ischemic/hypoxic conditions, via modulating vasodilation, endothelial leakage and anti-inflammatory responses (Takahashi et al., 2010). Adenosine levels increase rapidly with tissue hypoxia and inflammation (Rivkees and Wendler, 2011) and act as neuromodulator conferring both protective and deleterious effects via the activation of different adenosine receptors (e.g., A1, A2a, A2b, and A3) during several pathological conditions (Levy et al., 2004; Rivkees and Wendler, 2011; Farr et al., 2020). Whether increases of blood purines stem from the brain or are due to peripheral release or whether the detected increases represent a combination of both, remains to be established. Due to the high purine quantities detected in the circulation post-NE, contributions of brain-derived purines is, however, unlikely under mild NE and may be restricted to severe cases of NE with the associated brain injury. Two possible peripheral sources of purine release during and following NE include muscle tissue following increased muscle activity and systemic inflammation. While muscle activity is an unlikely source, as neonates with and without NE present similar behaviors [e.g., neonatal seizures are often accompanied by only subtle changes in behavior, further complicating their diagnosis (Murray et al., 2016)], there is a substantial body of evidence demonstrating altered immune responses (e.g., neutrophil activation) post-NE possibly contributing to increased purine/adenosine concentrations (Barletta et al., 2012; Karmakar et al., 2016; Wang and Chen, 2018; O’Dea et al., 2020; Kelly et al., 2021). Of note, similar to increased blood purines in infants with seizures, inflammation markers are also highly associated with seizures (Zareen et al., 2020). Nevertheless, the source of blood purine changes should be addressed in future studies.

Because our approach provides a combined measurement of adenosine and the adenosine breakdown products inosine, hypoxanthine and xanthine, we are unable to determine which of these purines contributes to the observed increase in blood purine concentrations during NE. However, due to the longer half-live (minutes) of inosine and hypoxanthine when compared to adenosine (seconds), these are most likely adenosine down-stream purines (Wung and Howell, 1984; Moser et al., 1989). In line with this, previous studies have shown increased hypoxanthine and xanthine in the blood following hypoxia in neonates (Saugstad, 1975; Harkness et al., 1983; Buonocore et al., 2000; Plank et al., 2011).

It is, however, important to keep in mind that changes in blood purine concentrations are not unique to hypoxia and NE and have been previously reported following traumatic brain injury, cerebral and cardiac ischemia, endotoxemia, and seizures among others (Weigand et al., 1999; Laghi Pasini et al., 2000; Bell et al., 2001; Ramakers et al., 2011; Farthing et al., 2015; Dale et al., 2019; Beamer et al., 2021). Blood adenosine concentrations have also been found increased in umbilical cord blood in infants following vaginal deliveries when compared to elective cesarean sections (Irestedt et al., 1989) and in premature newborns with white matter brain injury (Panfoli et al., 2016, 2018). Thus, elevations in blood purines/adenosine levels may represent a global phenomenon in response to any type of brain injury, rather than being specific to hypoxia or NE. Nevertheless, a biomarker for NE would most likely not be used as a stand-alone test and would be evaluated within a clinical context in combination with other measures. Moreover, while diagnostic tools for the identification of NE are available, there is a pressing need to stratify infants by severity using biomarkers. This would identify neonates with mild NE, which do not meet diagnostic criteria for TH but who have been shown to have adverse long-term neurodevelopment outcomes if untreated (Finder et al., 2020).

Although one of the main limitations of the study is the small sample size, infants were enrolled at two hospital sites for validation. Even though blood purine concentrations seem to correlate with seizures, infant numbers will have to be significantly increased to validate these results. Finally, purine measurements should be taken at different time-points during longitudinal sampling and purine concentrations correlated to response to treatment and clinical outcomes and other confounding factors such as duration of labor, vaginal vs. cesarean birth and elective vs. emergency delivery. Moreover, although sampling was carried out as early as possible within the first 4 days post-delivery, next steps should confirm higher blood purine levels post-NE immediately after birth.

In summary, this study demonstrates that blood purine nucleoside concentrations are increased following NE and have potential to be a novel bedside test based on blood purine changes. This may provide another tool for clinicians for a timely diagnosis of NE, critical for improving clinical outcomes.

## Data Availability Statement

The raw data supporting the conclusions of this article will be made available by the authors, without undue reservation.

## Ethics Statement

The studies involving human participants were reviewed and approved by the Ethics Committee of the Coombe Women and Infants University Hospital (CWIUH), Dublin, Ireland and Clinical Research Ethics Committee of the Cork Teaching Hospitals (CUMH) (ECM 5(5) 04/07/17). Written informed consent to participate in this study was provided by the participants’ legal guardian/next of kin. The animal study was reviewed and approved by Research Ethics Committee of the Royal College of Surgeons in Ireland (RCSI) (REC1302b) under license from the Department of Health, Dublin, Ireland (B100/4524).

## Author Contributions

EB measured blood purine levels in mice and edited parts of the manuscript. MO’D recruited infants and measured blood purine levels at CWIUH and edited manuscript. AG recruited infants and measured blood purine levels at CUMH and edited manuscript. JS performed experiments in hypoxic mouse pups and edited manuscript. AM-M analyzed patient data, edited the manuscript, and designed the figures. LK supported patient recruitment at CWIUH. AP supported patient recruitment at CUMH. SQ helped with analysis of data from hypoxic mouse model. MA analyzed data from mouse model. EJ-M and ED edited the manuscript. FT and ND edited the manuscript, designed and advised on use of SMARTChips, and helped with interpretation of data. GB, ED, and DM edited the manuscript and supported patient recruitment at CUMH. EM edited the manuscript, designed the experiments, and supported patient recruitment at CWIUH. TE designed the experiments and wrote the manuscript. All authors contributed to the article and approved the submitted version.

## Conflict of Interest

The authors declare that the research was conducted in the absence of any commercial or financial relationships that could be construed as a potential conflict of interest.

## Publisher’s Note

All claims expressed in this article are solely those of the authors and do not necessarily represent those of their affiliated organizations, or those of the publisher, the editors and the reviewers. Any product that may be evaluated in this article, or claim that may be made by its manufacturer, is not guaranteed or endorsed by the publisher.
